# Prevalence of secondary traumatic stress and its associated factors among oncology nurses: a multicenter cross-sectional study

**DOI:** 10.3389/fpubh.2026.1889359

**Published:** 2026-07-08

**Authors:** Li Wang, Xiaorong Xu, Xue Yang, Ting Wang

**Affiliations:** 1 Division of Biliary Tract Surgery, Department of General Surgery, West China Hospital, Sichuan University, Chengdu, Sichuan, China; 2 Department of Medical Oncology, Cancer Center, West China Hospital, Sichuan University, Chengdu, Sichuan, China; 3 Lung Cancer Center/Lung Cancer Institute, West China Hospital, Sichuan University, Chengdu, Sichuan, China

**Keywords:** oncology nurses, prevalence, secondary traumatic stress, self-regulatory fatigue, sleep quality

## Abstract

**Background:**

Although secondary traumatic stress (STS) is common in nurses and is associated with various psychological disorders such as anxiety and depression, the prevalence and its associated factors of STS specifically among oncology nurses remain unclear. This study aimed to determine the prevalence of STS and its associated factors among oncology nurses.

**Methods:**

A cross-sectional correlational study was conducted using convenience sampling to recruit nurses from five tertiary hospitals in Sichuan Province, China, between July and December 2025. Data collection tools included a demographic information questionnaire, the Secondary Traumatic Stress Scale, and the Pittsburgh Sleep Quality Index. Univariate analysis and binary logistic regression were employed to explore the factors associated with STS among oncology nurses.

**Results:**

Among the 536 enrolled oncology nurses, 342 (63.8%) were screened positive for STS symptoms based on the STSS threshold. Age (31–45 years: OR = 0.412, 95% CI: 0.181–0.929, *p* = 0.033; 46–60 years: OR = 0.315, 95% CI: 0.113–0.874, *p* = 0.026), educational background (undergraduate: OR = 0.637, 95% CI: 0.416–0.976, *p* = 0.038; postgraduate: OR = 0.505, 95% CI:0.467–0.851, *p* = 0.015), work experience (11–20 years: OR = 0.466, 95% CI: 0.351–0.658, *p* = 0.034; > 20 years: OR = 0.319, 95% CI:0.268–0.545, *p* = 0.017), night shift (OR = 1.752, 95% CI: 1.289–2.563, *p* = 0.035), sleep quality (OR = 2.354, 95% CI: 2.014–3.513, *p* < 0.001), and self-regulatory fatigue (OR = 1.647, 95% CI: 1.157–2.256, *p* < 0.001) were identified as significantly associated factors of STS among oncology nurses.

**Conclusion:**

Our findings revealed that STS was highly prevalent among oncology nurses and was significantly correlated with age, educational background, work experience, night shift, sleep quality, and self-regulatory fatigue. These findings suggest that nursing managers should prioritize STS prevention by providing individualized psychosocial interventions, such as mindfulness training, resilience cultivation, peer support, and sleep hygiene education.

## 1 Introduction

Secondary traumatic stress (STS) is defined as the adverse behaviors and emotional experiences arising from awareness of or exposure to another person’s traumatic experience, characterized by intrusion, avoidance, and hyperarousal symptoms (1). STS is prevalent among nurses and has been significantly associated with multiple adverse psychological and organizational outcomes (2). Previous studies have demonstrated that STS is related to anxiety, depression, burnout, compassion fatigue, and suicidal ideation in nurses (2, 3). Furthermore, STS may indirectly contribute to increased medication errors and reduced quality of care, thereby posing a serious threat to clinical patient safety (4, 5). It is worth noting that, compared with nurses in other specialties, oncology nurses are a more susceptible population to STS (6, 7). First, oncology nurses generally establish long-term therapeutic relationships with cancer patients. This sustained nurse–patient interaction makes them more likely to be drawn into the emotional experiences of patients, witnessing patients’ suffering, deterioration, and even death (8). Second, they often face high-intensity traumatic events, including delivering poor prognoses, witnessing severe chemotherapy-related complications, accompanying dying patients, and comforting bereaved family members (9). However, despite the theoretical and clinical relevance of STS in oncology nursing, empirical data on its prevalence among oncology nurses remain limited, particularly from multicenter samples.

Studies conducted among general nursing populations have identified several potential factors associated with STS. In terms of demographic characteristics, gender, age, educational background, and years of working experience have been found to be associated with STS among nurses (10, 11). Regarding work-related factors, night shift work and lack of social support have also been demonstrated to exacerbate STS symptoms in nurses (12). However, evidence on the specific factors contributing to STS in oncology nurses remains limited. Addressing this gap warrants the application of a well-established theoretical framework to clarify the underlying mechanisms. Drawing on the Conservation of Resources (COR) theory, individuals are generally motivated to protect and acquire valued resources, as psychological distress arises precisely when such resources are lost or inadequately replenished (13). Within this theoretical framework, sleep quality and self-regulatory fatigue can be conceptualized as critical components along the resource loss and gain continuum. Sleep quality constitutes a restorative resource. Adequate sleep facilitates the recovery of physical and psychological resources expended during work, whereas poor sleep quality reflects insufficient resource restoration and hastens resource depletion (14). Oncology nurses are repeatedly exposed to patient suffering and death, and poor sleep quality may disrupt the recovery process and further aggravate resource loss. Self-regulatory fatigue refers to a temporary decline in self-control and emotional regulation capacity (15). From a COR theory perspective, it represents a depleted state arising from cumulative resource loss and insufficient restoration. Oncology nurses constantly regulate their emotions and maintain professional composure, a process that consumes significant self-regulatory resources. When chronic depletion occurs without adequate replenishment, they are likely to develop self-regulatory fatigue, thereby impairing their ability to cope with STS. Therefore, the COR theory provides a coherent theoretical lens through which to understand the extent to which sleep quality and self-regulatory fatigue influence STS in oncology nurses. Specifically, we hypothesized that poor sleep quality and higher levels of self-regulatory fatigue would be positively associated with STS among oncology nurses.

In summary, the literature indicates that although oncology nurses are a susceptible population for STS, existing research has mainly focused on general clinical nurses, with limited attention given to the oncology setting. Furthermore, few studies have simultaneously examined the associations of sleep quality and self-regulatory fatigue with STS among oncology nurses within a unified theoretical framework. Moreover, most existing evidence has been generated in Western healthcare settings, and the applicability of these findings to the Chinese cultural context remains unclear. These limitations hinder a comprehensive understanding of the prevalence and associated factors of STS in oncology nurses, thereby potentially precluding the design and implementation of adequate organizational strategies designed to mitigate this condition. Therefore, grounded in the COR theory, this study aimed to examine the prevalence of STS and its associated factors among oncology nurses.

## 2 Methods

### 2.1 Study design, setting, and participants

A multicenter cross-sectional design was conducted, employing convenience sampling to recruit oncology nurses from five tertiary hospitals in Sichuan Province, China, between July and December 2025. Eligible participants were required to hold a valid nurse practicing certificate, work in an oncology department, and provide direct clinical care to oncology patients. Exclusion criteria included nurses who were pregnant, ill, or on personal leave during the data collection period, as well as those in managerial roles.

*A priori* sample size calculation was conducted using PASS software. Based on a meta-analysis reporting a pooled prevalence of 66.84% among oncology nurses (6), we assumed a 95% confidence level, an anticipated proportion (P) of 0.6684, and a two-sided confidence interval width of 0.10. The minimum required sample size was calculated to be 340 participants.

### 2.2 Instruments

#### 2.2.1 Demographic information questionnaire

The demographic information questionnaire included eight variables, including gender, age, marital status, educational background, work experience, monthly income (CNY), technical title, and night shift.

#### 2.2.2 Secondary traumatic stress scale (STSS)

The STSS is used to measure STS among oncology nurses (16). The 17-item instrument comprises three dimensions: intrusion, avoidance, and arousal. Each item is scored from 1 (never) to 5 (very frequently), with total scores ranging from 17 to 85, where higher scores indicate a greater frequency of STS symptoms. Severity categories of STS were defined based on the total score as follows: little or none (≤ 27), mild (28–37), moderate (38–43), high (44–48), and severe (≥ 49). In this study, the STSS achieved a good internal consistency, with a Cronbach’s *α* of 0.819.

#### 2.2.3 Pittsburgh sleep quality index (PSQI)

The PSQI is employed to assess sleep quality among oncology nurses (17). This 19-item scale evaluates sleep quality across seven dimensions: subjective sleep quality, sleep latency, sleep duration, sleep efficiency, sleep disturbances, use of sleep medication, and daytime dysfunction. Each item is rated on a Likert 4-point scale ranging from 0 (no difficulty) to 3 (severe difficulty). Total scores on the scale range from 0 to 21, with higher scores indicating poorer sleep quality. A cutoff score of > 5 is routinely used to define poor sleep quality. In this study, the Cronbach’s *α* of PSQI was 0.831.

#### 2.2.4 Self-regulatory fatigue scale (SRF-S)

Self-regulatory fatigue among oncology nurses was assessed using the SRF-S (18). The 16-item SRF-S uses a 5-point Likert scale (1 = strongly disagree, 5 = strongly agree) across three dimensions: cognitive control, emotional control, and behavioral control. Total scores of SRF-S range from 16 to 80, with higher values indicating greater self-regulatory fatigue. The Cronbach’s *α* for this scale was 0.825 in the present study.

### 2.3 Data collection

**Figure 1:**
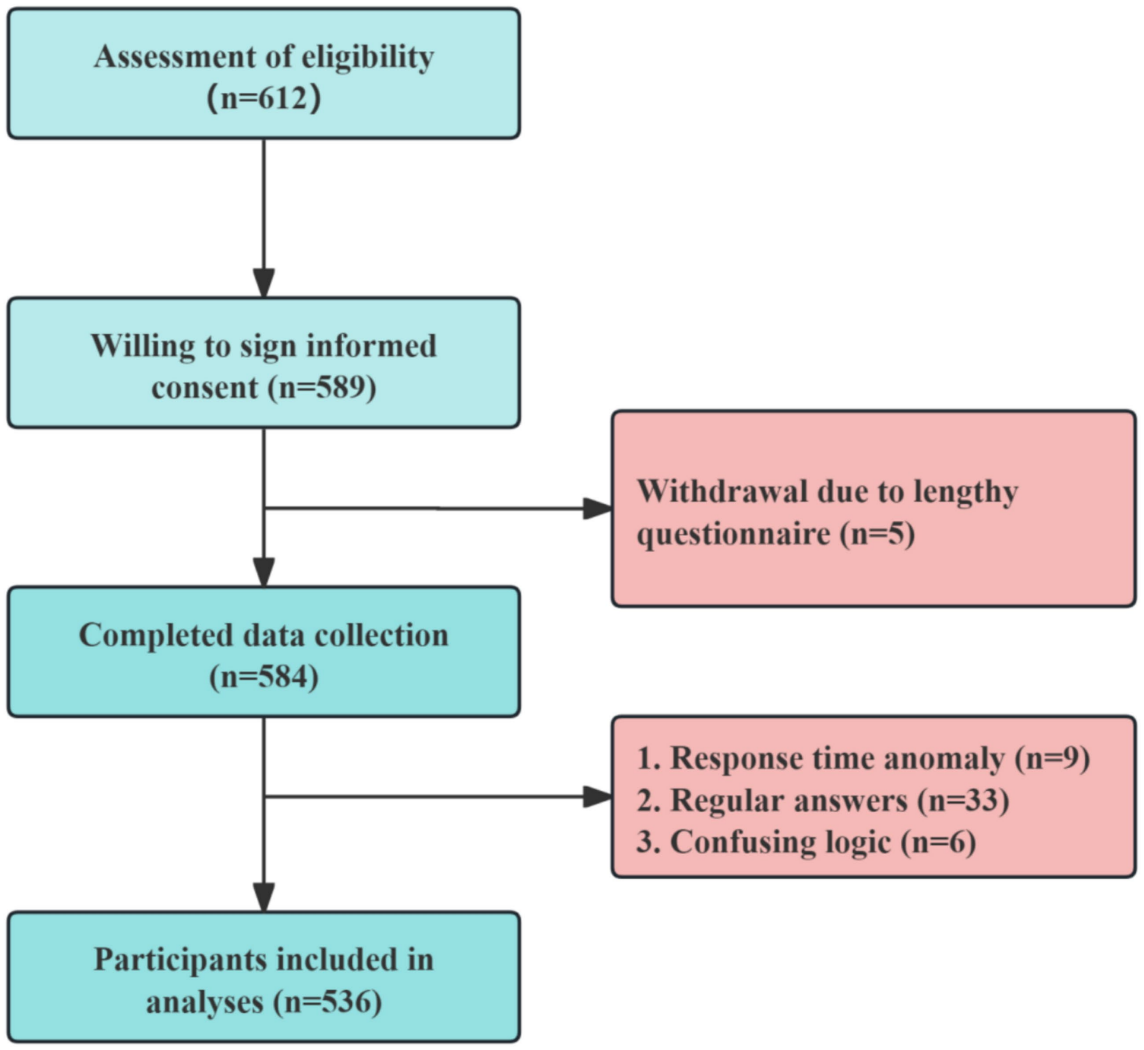
Participant enrollment process with screening outcomes.

Data collection was conducted using the Questionnaire Star platform. The research team collaborated with the nursing department of each hospital, which then distributed the survey link and QR code to nurses through head nurses via WeChat. Eligible participants were invited to complete an electronic survey voluntarily. The recruitment notice clearly stated the study purpose, significance, and precautions, emphasized that participation was entirely voluntary, and assured that refusal or withdrawal would not affect employment, performance evaluation, or professional relationships. Participants provided electronic informed consent before accessing the questionnaire. To ensure data quality, we restricted each IP address to one response, mandated all items, and enforced a minimum completion time of 8 min. Finally, the anonymous survey data were screened by two researchers, and questionnaires with abnormally long or short completion times or invariant response patterns were excluded. A total of 612 eligible nurses were identified across the five tertiary hospitals (Hospital A: *n* = 156; Hospital B: *n* = 120; Hospital C: *n* = 103; Hospital D: *n* = 125; Hospital E: *n* = 108). Of these, 584 questionnaires were distributed, yielding 536 valid returns and a response rate of 91.8%, with the participant enrollment process detailed in Figure 1.

### 2.4 Data analysis

Data analysis was conducted using SPSS 27.0. Continuous data were summarized as mean and standard deviation, and categorical data as frequencies and percentages. The Kolmogorov–Smirnov test was used to test the normality of continuous variables, and Levene’s test was used to assess homogeneity of variances between groups. Parametric tests were employed because the data met the assumptions required for these analyses. Chi-square tests and independent samples t-test were employed to examine differences between participants with and without STS. In addition, a binary logistic regression was performed to identify factors associated with STS. Multicollinearity across the variables was assessed using the variance inflation factor (VIF), with VIF > 5 considered indicative of significant collinearity. Model calibration was assessed using the Hosmer-Lemeshow goodness-of-fit test. *p* < 0.05 was considered statistically significant (two-sided).

### 2.5 Ethical considerations

Ethical approval in this study was obtained from the Ethics Committee of West China Hospital, Sichuan University (2025031). The study adhered to the Declaration of Helsinki. All participants gave informed consent, and their data were kept confidential.

## 3 Results

### 3.1 Demographic characteristics of participants

A total of 536 oncology nurses were included in the analysis. The sample was predominantly female (95.3%), with the largest age group being 31–45 years (50.0%). Moreover, most participants were married (57.5%) and held a bachelor’s degree (57.8%). Monthly income was primarily in the 5,000–10,000 CNY range (51.9%), and the majority had 1–10 years of work experience (45.7%). Finally, intermediate technical titles were most common (45.7%), and night shifts were reported by 68.1% of the nurses. The demographic characteristics of participants are summarized in Table 1.

**Table 1:** Demographic characteristics of participants and univariate analysis results.

Variables	Total sample (*n* = 536)	STS (*n* = 342)	Non-STS (*n* = 194)	*p*
Gender				0.156
Male	25 (4.7%)	14 (4.1%)	11 (5.7%)	
Female	511 (95.3%)	328 (95.9%)	183 (94.3%)	
Age (years)				< 0.001
18–30	188 (35.1%)	142 (41.5%)	46 (23.7%)	
31–45	268 (50.0%)	158 (46.2%)	110 (56.7%)	
46–60	80 (14.9%)	42 (12.3%)	38 (19.6%)	
Marital status				0.082
Unmarried	195 (36.4%)	135 (39.5%)	60 (30.9%)	
Married	308 (57.5%)	185 (54.1%)	123 (63.4%)	
Divorced/widowed	33 (6.2%)	22 (6.4%)	11 (5.7%)	
Educational background				< 0.001
Junior college	156 (29.1%)	118 (34.5%)	38 (19.6%)	
Undergraduate	310 (57.8%)	190 (55.6%)	120 (61.9%)	
Postgraduate	70 (13.1%)	34 (9.9%)	36 (18.6%)	
Monthly income (CNY)				0.065
< 5,000	165 (30.8%)	115 (33.6%)	50 (25.8%)	
5,000–10,000	278 (51.9%)	170 (49.7%)	108 (55.7%)	
> 10,000	93 (17.4%)	57 (16.7%)	36 (18.6%)	
Work experience (years)				< 0.001
1–10	245 (45.7%)	178 (52.0%)	67 (34.5%)	
11–20	210 (39.2%)	120 (35.1%)	90 (46.4%)	
>20	81 (15.1%)	44 (12.9%)	37 (19.1%)	
Technical title				0.124
Junior	198 (36.9%)	138 (40.4%)	60 (30.9%)	
Intermediate	245 (45.7%)	148 (43.3%)	97 (50.0%)	
Senior	93 (17.4%)	56 (16.4%)	37 (19.1%)	
Night shift				< 0.001
No	171 (31.9%)	84 (24.6%)	87 (44.8%)	
Yes	365 (68.1%)	258 (75.4%)	107 (55.2%)	
Sleep quality				< 0.001
Good sleep quality	216 (40.3%)	97 (28.4%)	119 (61.3%)	
Poor sleep quality	320 (59.7%)	245 (71.6%)	75 (38.7%)	
Self-regulatory fatigue	68.42 ± 12.56	74.28 ± 10.34	58.36 ± 9.87	< 0.001

### 3.2 Prevalence of STS among oncology nurses

**Figure 2:**
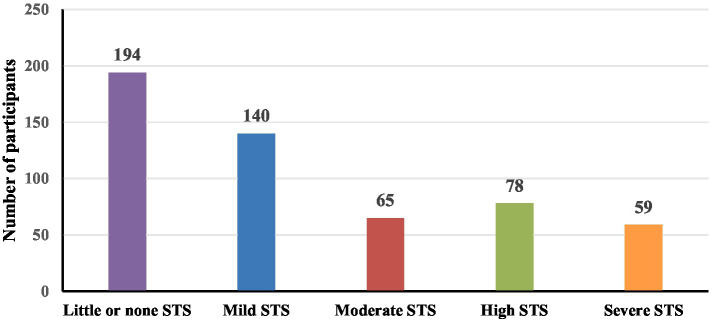
Prevalence of STS among oncology nurses.

Among 536 oncology nurses, 342 (63.8%) were screened positive for STS symptoms based on the STSS threshold, including 140 mild (40.9%), 65 moderate (19.0%), 78 high (22.8%), and 59 severe (17.3%). The prevalence of STS symptoms among oncology nurses is presented in Figure 2.

### 3.3 Univariate analysis of STS among oncology nurses

Demographic characteristics of oncology nurses with and without STS were compared using univariate analysis (Table 1). The univariate analysis revealed significant differences between oncology nurses with STS and those without STS in age, educational background, work experience, night shift, sleep quality, and self-regulatory fatigue (all *p* < 0.001), but no significant differences in gender (*p* = 0.156), marital status (*p* = 0.082), monthly income (*p* = 0.065), or technical title (*p* = 0.124).

### 3.4 Binary logistic regression analysis of STS among oncology nurses

Binary logistic regression was conducted to explore the associated factors of STS (Table 2). Participants with a total STSS score ≥ 28 were classified as having STS symptoms, while those with a score < 28 were classified as non-STS. For binary logistic regression, the dependent variable was secondary traumatic stress, and the independent variables (all *p* < 0.05 in univariate analysis) included age, educational background, work experience, night shift, sleep quality, and self-regulatory fatigue. Dummy variables were created for categorical variables, with the first category of each variable serving as the reference group. The variable assignment is presented in Table 3. Age (31–45 years: OR = 0.412, 95% CI: 0.181–0.929, *p* = 0.033; 46–60 years: OR = 0.315, 95% CI:0.113–0.874, *p* = 0.026), educational background (undergraduate: OR = 0.637, 95% CI: 0.416–0.976, *p* = 0.038; Postgraduate: OR = 0.505, 95% CI: 0.467–0.851, *p* = 0.015), work experience (11–20 years: OR = 0.466, 95% CI: 0.351–0.658, *p* = 0.034; > 20 years: OR = 0.319, 95% CI:0.268–0.545, *p* = 0.017), night shift (OR = 1.752, 95% CI: 1.289–2.563, *p* = 0.035), sleep quality (OR = 2.354, 95% CI: 2.014–3.513, *p* < 0.001), and self-regulatory fatigue (OR = 1.647, 95% CI: 1.157–2.256, *p* < 0.001) were identified as significantly associated factors of STS among oncology nurses. Multicollinearity diagnostics indicated that VIF values for all independent variables ranged from 1.05 to 3.52, suggesting no significant collinearity among the independent variables. The Hosmer-Lemeshow test yielded a χ^2^ of 6.73 (*p* = 0.566), indicating good model calibration.

**Table 2:** Binary logistic regression analysis of STS among oncology nurses.

Variables	β	SE	Wald	*p*	OR	95% CI	VIF
Constant	−2.845	1.520	3.502	0.061	0.058	-	
Age (years)							
18–30	Reference						3.52
31–45	−0.892	0.418	4.554	0.033	0.412	0.181–0.929	
46–60	−1.156	0.521	4.924	0.026	0.315	0.113–0.874	
Educational background							
Junior college	Reference						1.45
Undergraduate	−0.451	0.218	4.282	0.038	0.637	0.416–0.976	
Postgraduate	−0.683	0.266	6.596	0.015	0.505	0.467–0.851	
Work experience (years)							
1–10	Reference						3.41
11–20	−0.763	0.368	4.301	0.034	0.466	0.351–0.658	
>20	−1.142	0.479	5.682	0.017	0.319	0.268–0.545	
Night shift							1.12
No	Reference						
Yes	0.561	0.285	3.874	0.035	1.752	1.289–3.023	
Sleep quality							1.08
Good sleep quality	Reference						
Poor sleep quality	0.856	0.305	18.112	< 0.001	2.354	2.014–3.513	
Self-regulatory fatigue	0.499	0.036	19.266	< 0.001	1.647	1.157–2.256	1.05

**Table 3:** Binary logistic regression analysis variable assignment.

Variables	Assignment
Dependent variable	
Secondary traumatic stress	No = 0, Yes = 1
Independent variable	
Age (years)	18–30 = 1, 31–45 = 2, 46–60 = 3
Educational background	Junior college = 1, Undergraduate = 2, Postgraduate = 3
Work experience	1–10 = 1, 11–20 = 2, >20 = 3
Night shift	No = 0, Yes = 1
Sleep quality	Good sleep quality = 0, Poor sleep quality = 1
Self-regulatory fatigue	Continuous (mean score)

## 4 Discussion

This study aimed to determine the prevalence of STS and its associated factors among oncology nurses. The results revealed that 63.8% of oncology nurses were diagnosed with STS, and the prevalence was significantly associated with age, educational background, work experience, night shift, sleep quality, and self-regulatory fatigue. These findings provide an empirical basis for the development of comprehensive organizational management strategies at both the institutional and individual levels, with the goal of effectively reducing the prevalence of STS among oncology nurses and fostering a supportive clinical work climate.

Our study found that 63.8% of oncology nurses were screened positive for STS symptoms based on the STSS threshold, which is comparable to the rates observed in earlier investigations (6, 7). Cancer patients generally endure extended disease courses, repeated hospital admissions, and substantial physical and psychological suffering, manifested as pain, fear of recurrence, hopelessness, and existential distress (19, 20). Through sustained exposure to these profound emotional and existential challenges, oncology nurses experience a gradual erosion of psychological resilience, which in turn heightens their vulnerability to STS (21). Moreover, unlike other healthcare settings, oncology nursing involves substantial emotional demands, encompassing complex symptom management, disclosure of unfavorable news, facilitation of discussions about prognosis and advance care planning, and provision of bereavement support to family members (22). The execution of these emotionally taxing responsibilities under high workload and time constraints offers scant opportunity for emotional restoration, consequently increasing nurses’ susceptibility to STS (23).

In our study, age, educational background, work experience, and night shift were identified as factors associated with STS among oncology nurses. Specifically, older oncology nurses who had higher educational levels and longer work experience were less likely to suffer from STS. In contrast, those engaged in night shift work were at a higher risk of developing STS. First, younger oncology nurses with less work experience generally lack the requisite emotional coping strategies to handle the high psychological demands inherent in oncology nursing (24). Conversely, older nurses demonstrate more robust emotional regulation, allowing them to more effectively anticipate and navigate emotionally challenging situations, thereby shielding themselves from STS. Second, nurses with higher education benefit from comprehensive training in psychosocial support to cope with STS. Furthermore, higher education is linked to greater professional autonomy, better career prospects, and easier access to psychological support services, all of which protect against STS (8). Finally, night shift work contributes to a more stressful environment, marked by diminished staffing and insufficient immediate support. This heightened stress exposure predisposes nurses to adverse psychological outcomes, including burnout, anxiety, and depression, all of which have been directly implicated in the onset of STS (25).

Our findings demonstrated a significant association between sleep quality and STS among oncology nurses. Specifically, oncology nurses reporting poor sleep quality exhibited a higher risk of STS compared with those with good sleep quality. Poor sleep quality is highly prevalent among nurses and has been linked to psychological disorders such as anxiety and depression, both of which are recognized risk factors for STS (26). In addition, previous studies have shown that sleep deprivation impairs the brain’s capacity to process negative emotions and weakens prefrontal control over the amygdala, resulting in heightened emotional reactivity and reduced ability to manage work-related stress (27). As a result, oncology nurses with poor sleep quality are less capable of regulating their emotional responses to patient suffering, trauma, and death, thereby increasing their susceptibility to STS (28). Finally, from a resource conservation perspective, sleep is a critical energy resource. While restorative sleep restores the emotional and cognitive resources expended at work, poor sleep quality diminishes this resource pool, leaving nurses less equipped to handle daily stressors (29).

Our findings indicated that self-regulatory fatigue was significantly correlated with STS among oncology nurses. Self-regulatory fatigue refers to a state of depletion of an individual’s self-control resources (30). Oncology nurses continuously invest their resources to suppress negative emotions, maintain a professional demeanor, manage empathic responses, and cope with the moral distress arising from patient suffering and death (15). According to the Conservation of Resources theory, resource depletion occurs when resource investment fails to yield adequate returns or when resources are continuously consumed without sufficient replenishment (31). This depletion manifests as self-regulatory fatigue, which impairs nurses’ ability to regulate their emotional responses to work-related stressors, thereby increasing their vulnerability to STS (32). Moreover, self-regulatory fatigue may set in motion a downward resource loss spiral, in which depleted nurses are less capable of engaging in resource-replenishing activities such as effective self-care and seeking social support, resulting in compounded resource loss and more rapid progression toward STS (33).

### 4.1 Implications for nursing management

The findings of this study suggest several potential implications for nursing management aimed at addressing STS symptoms among oncology nurses. First, given that younger oncology nurses with shorter work experience and lower educational levels were more vulnerable to STS symptoms, nursing managers may consider developing targeted support programs for them, such as mentorship programs pairing junior nurses with experienced mentors, workshops focusing on emotional regulation, and coping strategies (34). Second, regarding night shift work, hospital administrators might consider optimizing shift scheduling by limiting consecutive night shifts and maintaining adequate staffing during night hours to allow sufficient recovery time between shifts, which may help reduce psychological stress among oncology nurses. Third, active measures to promote healthy sleep among oncology nurses, including sleep hygiene education and flexible scheduling aligned with individual circadian rhythms, could be considered (35). Fourth, at the organizational level, psychosocial interventions, including mindfulness training and peer support programs, may be implemented to assist oncology nurses in restoring depleted self-control resources and strengthening their capacity to withstand occupational stressors (36). However, it is important to note that these recommendations are derived from previous literature and theoretical considerations rather than from direct testing in the present study. Given the cross-sectional design of our study, future longitudinal or interventional research is needed to evaluate the effectiveness of these strategies in reducing STS symptoms among oncology nurses.

## 5 Limitations

This study has several limitations. First, the cross-sectional design limits any causal inferences about the relationships between the independent variables examined in this study and STS among oncology nurses. Second, convenience sampling was used to recruit participants from five tertiary hospitals in Sichuan Province, which may limit the representativeness of the sample and the generalizability of our findings to oncology nurses in other regions or healthcare systems. Finally, the assessments of all variables among oncology nurses were based on self-report questionnaires, which may have introduced recall and reporting biases.

## 6 Conclusion

In conclusion, STS was highly prevalent among oncology nurses and was significantly associated with age, educational background, work experience, night shift, sleep quality, and self-regulatory fatigue. These findings suggest that nursing managers should pay more attention to STS symptoms among oncology nurses. Individualized psychosocial interventions, including mindfulness training, resilience cultivation, peer support programs, and sleep hygiene education, may be beneficial, but their effectiveness in reducing STS risk needs to be confirmed in future longitudinal or interventional studies.

## Data Availability

The original contributions presented in the study are included in the article/supplementary material, further inquiries can be directed to the corresponding author.
